# Effect of prior antibiotic or chemotherapy treatment on immunotherapy response in non-small cell lung cancer

**DOI:** 10.1186/s12885-022-09210-2

**Published:** 2022-01-24

**Authors:** Andrew F. Nyein, Shahla Bari, Stephanie Hogue, Yayi Zhao, Bradley Maller, Sybil Sha, Maria F. Gomez, Dana E. Rollison, Lary A. Robinson

**Affiliations:** 1 Department of Cancer Epidemiology, Moffitt Cancer Center, Tampa, FL 33612 USA; 2 Division of Hematology & Oncology, Moffitt Cancer Center, Tampa, FL 33612 USA; 3 Department of Thoracic Oncology, Moffitt Cancer Center, 12902 Magnolia Drive, Tampa, FL 33612 USA; 4 Morsani College of Medicine, University of South Florida, Tampa, FL 33612 USA; 5 Cornell University, Ithaca, NY 14853 USA

**Keywords:** Non-small cell lung cancer, Immune checkpoint inhibitors, Immunotherapy, Antibiotics, Chemotherapy, Microbiome

## Abstract

**Background:**

Treatment outcomes of advanced non-small cell lung cancer (NSCLC) have substantially improved with immune checkpoint inhibitors (ICI), although only approximately 19% of patients respond to immunotherapy alone, increasing to 58% with the addition of chemotherapy. The gut microbiome has been recognized as a modulator of ICI response via its priming effect on the host immune response. Antibiotics as well as chemotherapy reduce gut microbial diversity, hence altering composition and function of the gut microbiome. Since the gut microbiome may modify ICI efficacy, we conducted a retrospective study evaluating the effects of prior antibiotic or chemotherapy use on NSCLC patient response to ICI.

**Methods:**

We retrospectively evaluated 256 NSCLC patients treated between 2011–2017 at Moffitt Cancer Center with ICI ± chemotherapy, examining the associations between prior antibiotic or chemotherapy use, overall response rate and survival. Relative risk regression using a log-link with combinatorial expectation maximization algorithm was performed to analyze differences in response between patients treated with antibiotics or chemotherapy versus patients who didn’t receive antibiotics or chemotherapy. Cox proportional hazards models were constructed to evaluate associations between risk factors and overall survival.

**Results:**

Only 46 (18% of 256) patients used antibiotics prior to and/or during ICI treatment, and 146 (57%) had prior chemotherapy. Antibiotic users were 8% more likely to have worse overall response rate (RR:1.08; CI:0.93–1.26; *p* = 0.321), as well as a 35% worse overall survival (HR:1.35; CI:0.91–2.02; *p* = 0.145), although results were not statistically significant. However, prior use of chemotherapy was significantly associated with poor ICI response (RR:1.24; CI:1.05–1.47; *p* = 0.013) and worse overall survival (HR:1.47; CI:1.07–2.03; *p* = 0.018).

**Conclusions:**

Patients receiving antibiotics prior to and/or during ICI therapy might experience worse treatment outcomes and survival than unexposed patients, although these associations were not statistically significant and hence warrant further prospective study. Prior chemotherapy significantly reduced ICI response and overall survival. Antibiotic or chemotherapy exposure may negatively impact ICI response, perhaps through disruption of the eubiotic gut microbiome.

## Background

Lung cancer represents 13% of all newly diagnosed cancers and is the leading cause of cancer-related death, with an overall 5-year survival rate of only 15% [1, 2]. Of the various histological subtypes, non-small cell lung cancer (NSCLC) represents the majority of lung cancers (80–85%), with treatment options ranging from radiation and chemotherapy to surgical resection [1, 3]. Although treatment of early-stage disease (stages IA to IIB) results in a 5-year survival rate of 49–83% [4], early detection can be challenging due to lack of biomarkers and a prolonged latency period [5, 6]. Nearly 50% of lung cancers are therefore diagnosed at advanced clinical stage [7], where the cure rate is markedly reduced [8, 9].

Immune checkpoint inhibitors (ICIs) have changed the treatment paradigm of NSCLC, markedly improving response rates and survival [10]. ICIs function by blocking important regulatory receptors, thus releasing the brake on immune cells and allowing immune-mediated tumor recognition and killing [11, 12]. Unfortunately, over 50% of patients may fail to respond to ICI-chemotherapy combination treatment [10]. Those who respond, however, may experience a response duration upwards of 3 years [13]. Identifying biomarkers and modulators of ICI response is therefore critically important to distinguish those who might receive treatment benefit from those who might not.

Recently, the gut microbiome has been studied as a potential mediator of ICI efficacy. The influence of the gut microbiota on ICI response has been established by fecal microbiota transplant (FMT), whereby FMT from responding patients improved ICI response when transplanted to germ-free mice compared to mice transplanted with non-responding patient stool [14]. In humans, recent studies in various cancers, including NSCLC, have identified higher microbial diversity and enrichment of *Bifidobacterium* [15], *Akkermansia* [16]*, Faecalibacterium* [14], and well-known short-chain fatty acid-producing bacteria [17] in ICI responders versus non-responders. Given recent evidence for the potential role of the gut microbiome in mediating ICI response, it follows that medications, including antibiotics (ATBs) and cytotoxic chemotherapy, that can alter the gut microbiome and lead to dysbiosis may impact ICI response. Indeed, recent evidence shows that when cancer patients receive ICI therapy and have been exposed to ATBs prior to treatment, they experience worse survival [18–21].

While numerous studies have evaluated the role of prior and concurrent ATBs on ICI response, results have been inconsistent, with conflicting reports ranging from no effect [22] to an unfavorable effect on progression free survival and/or overall survival (OS) [23]. Furthermore, most studies have evaluated only the effect of ATBs on ICI response, whereas the effect of pretreatment chemotherapy on ICI response has been overlooked. To explore the role of the gut microbiome in mediating ICI response, we conducted a retrospective study to evaluate the effects of pre-treatment and concurrent ATB as well as cytotoxic chemotherapy exposure on ICI response in patients with advanced NSCLC (stage III/IV).

## Methods

### Study population

Of the 381 patient medical records screened, 256 were deemed eligible with adequate follow-up data. Eligible patients included men and women ≥ 18 years of age diagnosed with primary stage III or IV NSCLC, adenocarcinoma or squamous cell carcinoma, treated with ICIs at Moffitt Cancer Center (MCC) between January 1, 2011 and March 31, 2017. ICI agents included anti-PD-1, anti-PD-L1, or anti-CTLA-4, alone or in combination with other agents such as targeted therapy or chemotherapy. Approval for this retrospective chart review was obtained from the MCC Scientific Review Committee and Chesapeake Institutional Review Board, Protocol MCC 19,162.

### Clinical data abstraction

Clinical data was abstracted and transcribed into a secure database. Data elements were defined according to standard definitions and finalized in consultation with clinical investigators. Second-level abstractions were performed on a random sample of 20% of the medical records to ensure an accurate and unbiased abstraction process. Discrepancies were discussed and resolved by consensus of the research team.

Recent ATB use was assessed for the period two months before and up to one month after the initial ICI dose, and patients were dichotomized as having received or not received ATBs in this three-month period around ICI initiation. The primary clinical outcome was clinician-assessed response [complete response (CR), partial response (PR), stable disease (SD), or progressive disease (PD)] using radiographic interpretations with the Response Evaluation Criteria in Solid Tumors (RECIST) version 1.1 [24]. The best clinical response recorded within six months of ICI start was abstracted and patients were categorized as responders (CR/PR) or non-responders (SD/PD). OS, defined as the time between start of ICI and death or censored at date of last follow-up, was examined as a secondary clinical outcome.

Sociodemographic characteristics such as sex, age, race/ethnicity, smoking status, as well as date of death or last contact were recorded. Indication for ATB use, duration, dose and type of ATB used were also recorded. Furthermore, prior chemotherapy within 6 months of ICI treatment, other medication usage, and comorbidities [evaluated and graded according to the Charlson Comorbidity Index (CCI)] were abstracted from the medical record.

### Statistical analyses

Baseline characteristics were reported and stratified by ATB use (primary exposure) and by clinician-assessed response (primary outcome). Wilcoxon rank-sum test and Chi-squared test were used to assess whether baseline characteristics were associated with the exposure and/or outcome. Treatment response was cross-tabulated with reason for ATB use, with p-values calculated using Fisher’s exact test. Mean and standard deviation of the duration and dose of ATB use were also reported.

Relative risk (RR) regression using a log-link with combinatorial expectation maximization (CEM) algorithm [25] was used to assess the association between recent ATB use and other risk factors with clinical response. In addition to univariate RR models, a multivariate model (multivariate 1) was constructed to assess the association between ATB use and clinical response, adjusting for baseline characteristics that were found to be associated with either exposure or outcome. Subsequently, a more inclusive model (multivariate 2) was constructed to include additional relevant factors, such as age, ECOG, and prior targeted therapy. Kaplan–Meier analyses were performed, and Cox proportional hazard models were constructed to examine the association between risk factors and OS. Adjusted Cox models incorporated relevant clinical variables, such as age, prior chemotherapy and ECOG performance status, determined by recursive stepwise selection. Statistical analyses were performed using R, version 4.0.4 (R Foundation for Statistical Computing, Vienna, Austria, 2019). P values < 0.05 were considered statistically significant.

## Results

### Patient characteristics

This retrospective analysis included 256 NSCLC patients. Mean patient age was 65 years (range 45–85 years), with a relatively equal number of men and women (136 men, 53%). The majority were white (241, 94%), married (156, 61%), and former smokers (184, 72%). Most (198, 77%) of these patients had ECOG performance status of 1–2 and 94% (240) were diagnosed with stage 4 disease. Fifty seven percent of patients (147) had adenocarcinoma, 23% (53) had squamous cell carcinoma and 20% (50) had other subtypes of NSCLC. Over half of patients (146, 57%) had received chemotherapy within six months of ICI treatment, 31% received radiation treatment and 16% received other targeted treatments prior to ICI therapy. Complete demographic and clinical data are shown in Table 1. Overall, there were no significant differences in baseline demographic and clinical patient characteristics between ATB users and non-users, except for the use of proton pump inhibitors (PPIs), which was significantly more common in patients unexposed to ATBs versus those who were exposed.

**Table 1:** Baseline characteristics by antibiotic exposure

		**Exposure**			**Outcome**		
Factors	**Total (*n* = 256)**	**ATB (-) Group (*n* = 210)**	**ATB ( +) Group (*n* = 46)**	***P*-value**	**CR & PR (*n* = 71)**	**SD & PD (*n* = 185)**	***P*-value**
Age (Years) Mean [SD]	65.5 [9.5]	65.5 [9.6]	65.3 [9.4]	0.942	66.5 [9.7]	65.1 [9.4]	0.400
Ethnicity- No. (%)							
Non-Hispanic	243 (94.9)	200 (95.2) 10	43 (93.5) 3	0.903	69 (97.2) 2	174 (94.1)	
Hispanic/Latino	13 (5.1)	(4.8)	(6.5)		(2.8)	11 (5.9)	0.482
Pack Years							
Mean [SD]	32.9 [25.9]	33.8 [26.3]	29.2 [23.9]	0.435	30.5 [23.9]	33.8 [26.6]	0.496
Clinical Stage- No. (%)							
Stage 3	16 (6.2)	14 (6.7)	2 (4.3)	0.801	5 (7.0)	11 (5.9)	0.971
Stage 4	240 (93.8)	196 (93.3)	44 (95.7)		66 (93.0)	174 (94.1)	
Race- No. (%)							
White	241 (94.2)	200 (95.2)	41 (89.1)		65 (91.5)	176 (95.1)	
Black or African American	9 (3.5)	7(3.3)	2 (4.3)	0.109	3 (4.2)	6 (3.2)	0.429
Asian	6 (2.3)	3(1.4)	3 (6.5)		3 (4.2)	3 (1.6)	
Gender- No. (%)							
Female	120 (47.0)	98 (46.7)	22 (47.8)	1.000	29 (40.8)	91 (49.2) 94	0.290
Male	136 (53.0)	112 (53.3)	24 (52.2)		42 (59.2)	(50.8)	
Smoking Status- No. (%)							
Never	38 (14.8)	29 (13.8)	9 (19.6)		10 (14.1)	28 (15.1)	
Former	184 (71.9)	153 (72.9)	31 (67.4)	0.606	51 (71.8)	133 (71.9)	0.958
Current	34 (13.3)	28 (13.3)	6 (13.0)		10 (14.1)	24 (13.0)	
Marital Status- No. (%)							
Single	23 (9.0)	19 (9.0)	4 (8.7)		10 (14.1)	13 (7.0)	
Married/Cohabitating	156 (60.9)	133 (63.3)	23 (50.0)	0.601	40 (56.3)	116 (62.7)	0.185
Widowed/Divorced/Separated	48 (18.8)	38 (18.1)	10 (21.7)		12 (16.9)	36 (19.5)	
Not Reported	29 (11.3)	20 (9.6)	9 (19.6)		9 (12.7)	20 (10.8)	
Prior Surgery- No. (%)							
No	203 (79.3)	162 (77.1)	41 (89.1)	0.106	59 (83.1)	144 (77.8)	0.449
Yes	53 (20.7)	48 (22.9)	5 (10.9)		12 (16.9)	41 (22.2)	
Prior Chemotherapy- No. (%)							
No	110 (43.0)	92 (43.8)	18 (39.1)	0.677	41 (57.7)	69 (37.3)	0.005^*^
Yes	146 (57.0)	118 (56.2)	28 (60.9)		30 (42.3)	116 (62.7)	
Prior Radiation- No. (%)							
No	178 (69.5)	148 (70.5)	30 (65.2)	0.600	50 (70.4)	128 (69.2)	0.968
Yes	78 (30.5)	62 (29.5)	16 (34.8)		21 (29.6)	57 (30.8)	
Prior Targeted Therapy- No. (%)							
No	215 (84.0)	173 (82.4)	42 (91.3)	0.203	64 (90.1)	151 (81.6)	0.141
Yes	41 (16.0)	37 (17.6)	4 (8.7)		7 (9.9)	34 (18.4)	
Histology- No. (%)							
Adenocarcinoma	147 (57.4)	119 (56.7)	28 (60.9)		43 (60.6)	104 (56.2)	
Squamous Cell Carcinoma	59 (23.0)	49 (23.3)	10 (21.7)	0.865	15 (21.1)	44 (23.8)	0.818
Other NSCLC	50 (19.6)	42 (20.0)	8 (17.4)		13 (18.3)	37 (20.0)	
NSAID Use- No. (%)							
No	235 (91.8)	190 (90.5)	45 (97.8)	0.177	63 (88.7)	172 (93.0)	0.394
Yes	21 (8.2)	20 (9.5)	1 (2.2)		8 (11.3)	13 (7.0)	
PPI Use- No. (%)							
No	195 (76.2)	153 (72.9)	42 (91.3)	0.014^*^	50 (70.4)	145 (78.4)	0.241
Yes	61 (23.8)	57 (27.1)	4 (8.7)		21 (29.6)	40 (21.6)	
ECOG- No. (%)							
0	57 (22.3)	50 (23.8)	7 (15.2)		18 (25.4)	39 (21.1)	
1	192 (75.0)	153 (72.9)	39 (84.8)	0.195	52 (73.2)	140 (75.7)	0.662
2	6 (2.3)	6 (2.9)	0 (0.0)		1 (1.4)	5 (2.7)	
Not Reported	1 (0.4)	1 (0.4)	0 (0.0)		0 (0.0)	1 (0.5)	
Charlson Comorbidity Index							
Mean [SD]	6.9 [1.6]	6.9 [1.6]	6.9 [1.5]	0.766	7.1 [1.7]	6.9 [1.5]	0.528

^*^Statistically Significant, when *p* < 0.05; Age, Pack Years, ECOG, and CCI (Wilcoxon Rank Sum Test); Other Variables (Chi-Square Analysis). Smoking variables not necessarily obtained at treatment start (electronic new patient questionnaire). *N/A* Not Applicable, *ATB* antibiotic, *PPI* Proton pump inhibitors, *NSAID* Non-steroidal anti-inflammatory drugs, *CR* Complete response, *PR*: Partial response, *PD* Progressive disease, *SD* Stable disease

### Antibiotic use

Of the total 256 patients, 46 (18%) received ATBs within 60 days of ICI initiation or concurrently with the first month of ICI therapy. Both oral and intravenous ATBs were used, including β-lactams, fluoroquinolones, macrolides, cephalosporins and tetracyclines. The three most prescribed ATBs were levofloxacin (*n* = 15), cefazolin (*n* = 14), and azithromycin (*n* = 8). ATBs were prescribed to patients for various indications, with the most frequent being prophylactic use prior to surgery (*n* = 15) and treatment of upper respiratory tract infections (*n* = 25), as shown in Table 2. The average duration of ATB use was approximately 1 day for surgical prophylaxis and 8 days for respiratory tract infection.

**Table 2:** Reasons for ATB use, by treatment outcome

	**Treatment response within 6 months**			**Duration (days)**^**2**^	**Dose (mg)**^**2**^
	**CR/PR (*n* = 12)**^**1**^	**SD/PD (*n* = 34)**	***P*-value**	**Mean [SD]**	**Mean [SD]**
Surgery					
No	8	23	1.000	1.12 [0.33]	1298.44 (657.04)
Yes	4	11			
RTI					
No	5	16	1.000	7.84 [6.43]	587.07 (590.89)
Yes	7	18			
Other Reasons					
No	7	20	1.000	13.65 [10.60]	375.24 (265.38)
Yes	5	14			

^*^Statistically Significant for Fisher’s exact test, when *p* < 0.05; 1: no CR among ATB users, only PR. 2: this is measured at the level of each ATB use, not at the individual patient-level. *CR* Complete response, *PR* Partial response, *PD* Progressive disease, *SD* Stable disease, *RTI* Respiratory tract infection, including bronchitis, pneumonia, upper respiratory tract infection, and sinusitis. Other reasons include patients taking antibiotics for acne, biopsy, cholangitis, colitis, ear cellulitis, leukocytosis, metastasis, rash, urinary tract infection, prophylaxis. Duration of antibiotic use for each reason was not conducted at patient-level, but instead calculated with antibiotic-use level analysis. Each patient may have overlapping antibiotic use; each use was treated as a single observation

### Treatment response

At 6 months following ICI start, 71 patients (28%) were considered ICI responders (CR/PR). The disease control rate was about two-thirds (66%), that is, 168 patients achieved a response of either CR, PR, or SD at 6 months post-ICI start. After adjusting for PPI use, prior chemotherapy, age, ECOG performance status and prior targeted therapy, relative risk analyses revealed that ATB-treated patients were 8% more likely to be ICI non-responders (SD/PD) compared to ATB-untreated patients (Table 3). However, this association did not reach statistical significance (RR: 1.08; CI:0.93–1.26; *p* = 0.321). At 12 months of follow up, results were not significantly different (data not shown). Additionally, there were no significant differences in treatment response based on the reasons for antibiotic use (Table 2).

**Table 3:** Relative risk of poor clinical response

	**Best Response in 6 months**						
	**CR/PR (*n* = 71)**	**SD/PD (*n* = 185)**	**Univariate**	**Multivariate**^**1**^		**Multivariate**^**2**^	
	***N* (%)**	***N* (%)**	**Relative Risk (95% CI)**	**Relative Risk (95% CI)**	***P*-value**	**Relative Risk (95% CI)**	***P*-value**
Antibiotic Use							
ATB-	59 (28.1)	151 (71.9)	1.00	1.00		1.00	
ATB +	12 (26.1)	34 (73.9)	1.03 (0.85–1.24)	1.04 (0.88–1.24)	0.633	1.08 (0.93–1.26)	0.321
PPI Use							
No	50 (25.6)	145 (74.4)	1.00	1.00		1.00	
Yes	21 (34.4)	40 (65.6)	1.00 (0.82–1.22)	1.00 (0.83–1.21)	1.000	1.00 (0.82–1.21)	1.000
Prior Chemotherapy							
No	41 (37.3)	69 (62.7)	1.00	1.00		1.00	
Yes	30 (20.5)	116 (79.5)	1.27 (1.07–1.50)	1.26 (1.07–1.48)	0.007*	1.24 (1.05–1.47)	0.013*
Age (binary)							
< = 67	39 (27.5)	103 (72.5)	1.00	NA	NA	1.00	
> 67	32 (28.1)	82 (71.9)	1.00 (0.86, 1.17)	NA	NA	1.00 (0.87–1.15)	1.000
ECOG Performance Status							
0	18 (31.6)	39 (68.4)	1.00	NA	NA	1.00	
1/2	53 (26.8)	145 (73.2)	1.07 (0.88–1.30)	NA	NA	1.06 (0.89–1.26)	0.530
Targeted Therapy							
No	64 (29.8)	151 (70.2)	1.00	NA	NA	1.00	
Yes	7 (17.1)	34 (82.9)	1.18 (1.00–1.39)	NA	NA	1.11 (0.95–1.29)	0.188

^*^Statistically Significant, when *p* < 0.05; 1: Adjusted for significant risk factors in Table 1/adjusted for PPI and Chemotherapy; 2: Adjusted for PPI, Chemotherapy, Age (cut at the median age of 67), ECOG (0 vs. combined 1/2), and Targeted Therapy; *Negative response ( SD&PD) is considered as event/1. Positive response (CR&PR) is considered as reference/0. *CR* complete response, *PP* Proton pump inhibitor, *PR* partial response, *PD* progressive disease, *SD* Stable disease

Interestingly, prior exposure to chemotherapy was significantly associated with an unfavorable ICI response (SD/PD) (RR: 1.24; CI: 1.05–1.47; *p* = 0.013) (Tables 1 and 3). However, the use of PPIs had no impact on treatment response at 6 months (Tables 1 and 3) or at 12 months (data not shown).

### Overall survival

**Fig.1:**
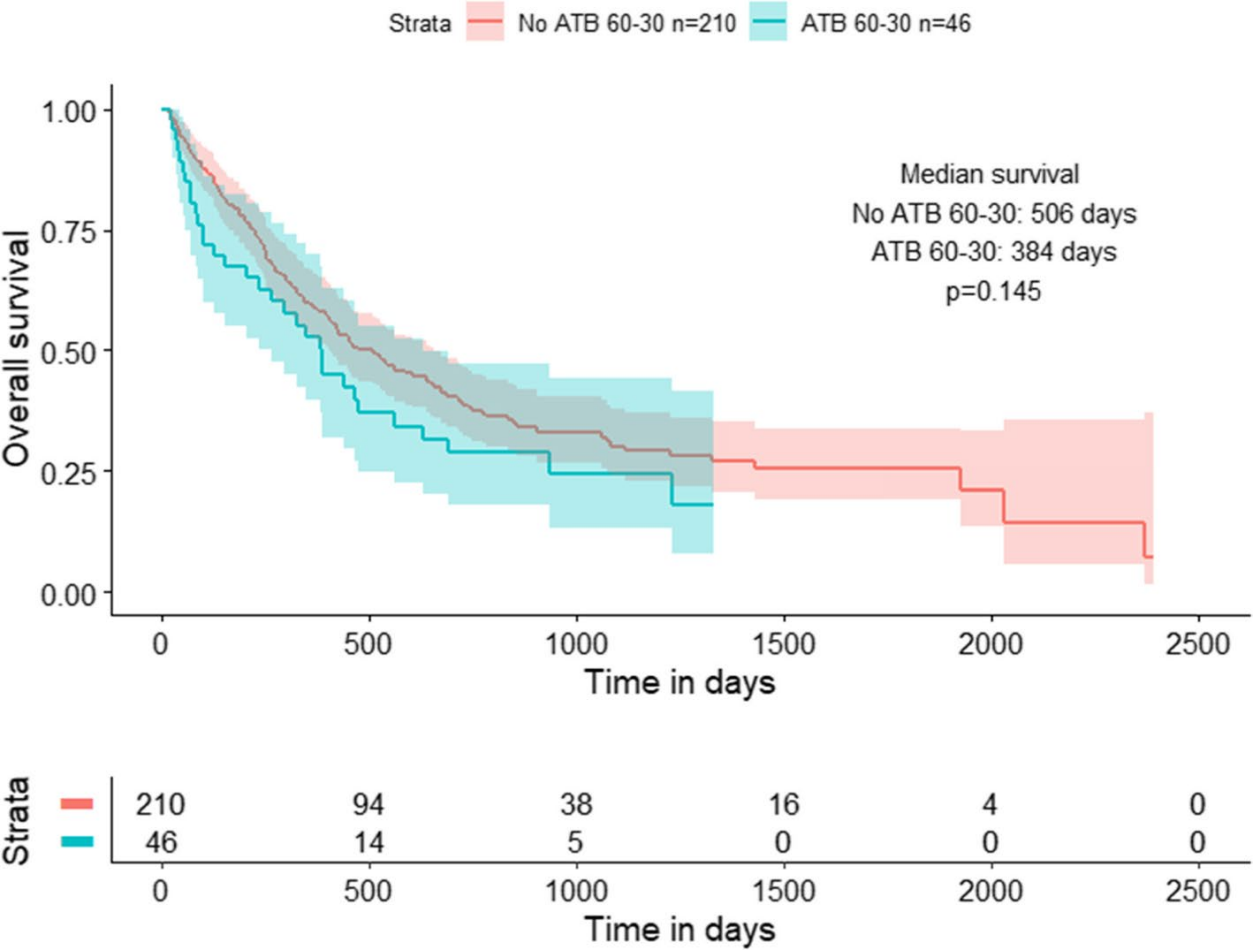
Kaplan Meier survival analysis for the effects of antibiotic use (60–30) on overall survival. Patients receiving antibiotics 60 days before to 30 days after the start of immunotherapy treatment experienced a diminished median survival of approximately 120 days compared to patients without prior exposure to antibiotics within the same time period

After adjusting the Cox proportional hazard model to account for potential confounding variables, including PPI use, prior treatment received, performance status and other factors, ATB use was associated with worse OS, however this was not statistically significant (HR:1.35;CI:0.91–2.02; *p* = 0.140) (Table 4). The Kaplan–Meier survival analysis further corroborated this finding, as the median OS was consistently higher among ATB-untreated versus ATB-treated patients, though the log-rank p-value was not statistically significant (Fig. 1). However, prior chemotherapy use (HR:1.47; CI:1.07–2.03; *p* = 0.018) and worse ECOG performance status (> 0) (HR: 1.75; CI: 1.19–2.57; *p* = 0.005) were associated with significantly worse OS.

**Table 4:** Cox proportional hazards model for overall survival

	**Alive (*n* = 84)**	**Deceased (*n* = 172)**	**Univariate**	**Multivariate**^**1**^	
	***N* (%)**	***N* (%)**	**Hazard Ratio** **(95% CI)**	**Hazard Ratio (95% CI)**	***P*-value**
Antibiotic Use					
ABT-	70 (83.3)	140 (81.4)	1.00	1.00	
ABT +	14 (16.7)	32 (18.6)	1.33 (0.91–1.96)	1.35 (0.91–2.02)	0.140
PPI Use					
No	62 (73.8)	133 (77.3)	1.00	1.00	
Yes	22 (26.2)	39 (22.7)	0.91 (0.63–1.29)	0.92 (0.64–1.33)	0.667
Prior Chemotherapy					
No	44 (52.4)	66 (38.4)	1.00	1.00	
Yes	40 (47.6)	106 (61.6)	1.42 (1.04–1.93)	1.47 (1.07–2.03)	0.018*
Age (mean [sd])					
NA	65.6 [10.1]	65.4 [9.2]	1.00 (0.99–1.02)	1.00 (0.98–1.02)	0.933
ECOG Performance Status					
0	24 (28.6)	33 (19.3)	1.00	1.00	
1/2	60 (71.4)	138 (80.7)	1.75 (1.19–2.57)	1.75 (1.19–2.57)	0.005*
Targeted Therapy					
No	73 (86.9)	142 (82.6)	1.00	1.00	
Yes	11 (13.1)	30 (17.4)	1.02 (0.69–1.52)	1.00 (0.66–1.52)	0.995
Smoking Status					
Never	13 (15.5)	25 (14.5)	1.00	1.00	
Former	57 (67.9)	127 (73.8)	1.30 (0.85–2.00)	1.36 (0.88–2.10)	0.168
Current	14 (16.7)	20 (11.6)	1.02 (0.57–1.84)	1.06 (0.58–1.92)	0.859

^*^Statistically Significant, when *p* < 0.05; 1: Adjusted for PPI, Chemotherapy, Age (Continuous), ECOG (0 vs. combined 1/2), Targeted Therapy, and Smoking Status; *Dead considered as event/1. Alive is reference/0. *PPI*: Proton pump inhibitor

## Discussion

We investigated the effect of prior and concurrent ATB use on ICI response and OS, specifically for anti-PD-1/anti-PDL-1 and anti-CTLA-4 immunotherapy treatment. Since stool samples were unavailable, ATB exposure was used as a proxy for gut microbial dysbiosis in these patients. After accounting for various confounders, ATB exposure was not statistically significantly associated with ICI treatment outcomes, although the trend suggested there may be an increased risk of poor outcomes in ATB users versus non-users. Prior chemotherapy, however, was significantly associated with worse response and overall survival. These results may suggest a potential role of medication-induced gut microbial dysbiosis in modulating immunotherapy response, which has been supported by a growing body of literature [20, 23, 26]. Specifically, the trillions of microorganisms that inhabit the gut play a critical role in priming our innate and adaptive immune response, including CD4 + /CD8 + T cells, which are vital for ICI response [11]. Dysbiosis, which includes altered diversity as well as an imbalance in the gut microbial community that might be induced by different medications, may lead to altered or ineffective CD4 + /CD8 + priming and, consequently, poor response to ICI [12].

Recent studies provide evidence that the effectiveness of anti-PD-L1 and anti-CTLA-4 blockade depends on the diversity of microbes as well as the presence of commensal *Bifidobacterium* and *Bacteroides* species, respectively [27, 28]. Investigators have recently demonstrated that replenishing the gut microbiome with *Akkermansia muciniphila* in germ-free mice receiving FMTs from patients responding to immunotherapy promotes CCR9 + CXCR3 + CD4 + T-lymphocyte migration into tumor beds [16]. These studies emphasize the importance of communication between the gut microbiota and the host’s immune system in mediating immunotherapeutic effects. Further mechanistic studies are needed to clarify the potential effects of ATBs on gut microbial dysbiosis in the context of ICI therapy.

This study also surprisingly demonstrated that prior chemotherapy predicted poor outcomes, with a 47% increased risk of death in those receiving prior chemotherapy compared to patients who did not. This detrimental effect of chemotherapy on ICI response and OS could be the results of two factors. First, chemotherapy is associated with immunosuppression characterized by delayed recovery of CD4 + T cells and B cells [29]. This dampened immune response may abrogate ICI response. Second, it has be shown that chemotherapy elicits gut dysbiosis and poor ICI response, perhaps via gastrointestinal mucositis [26, 30]. Other medications shown to impact the gut microbiome are PPIs, which may elicit dysbiosis via reduction of intragastric pH and thus selective pressure for distinct microbial populations [31]. PPI users have been shown to exhibit limited microbial diversity and increased susceptibility to enteric infections [31, 32]. However, in our study we did not find any significant association between PPI use and ICI response or OS. Similarly, another recent study found no effect of PPI use on efficacy of PD-1/PD-L1 blockade in epithelial cancers [33]. Future prospective research studies may provide further insight into the relationship between PPI use, the gut microbiome and ICI response.

### Strengths and limitations

This is a substantially large retrospective, single-institution study examining the effects of prior and concurrent ATBs on clinical response to ICI in patients with NSCLC. Although ATBs were not significantly associated with poor clinical outcomes, recent/concurrent ATB use was consistently associated with poorer prognoses.

**Fig. 2:**
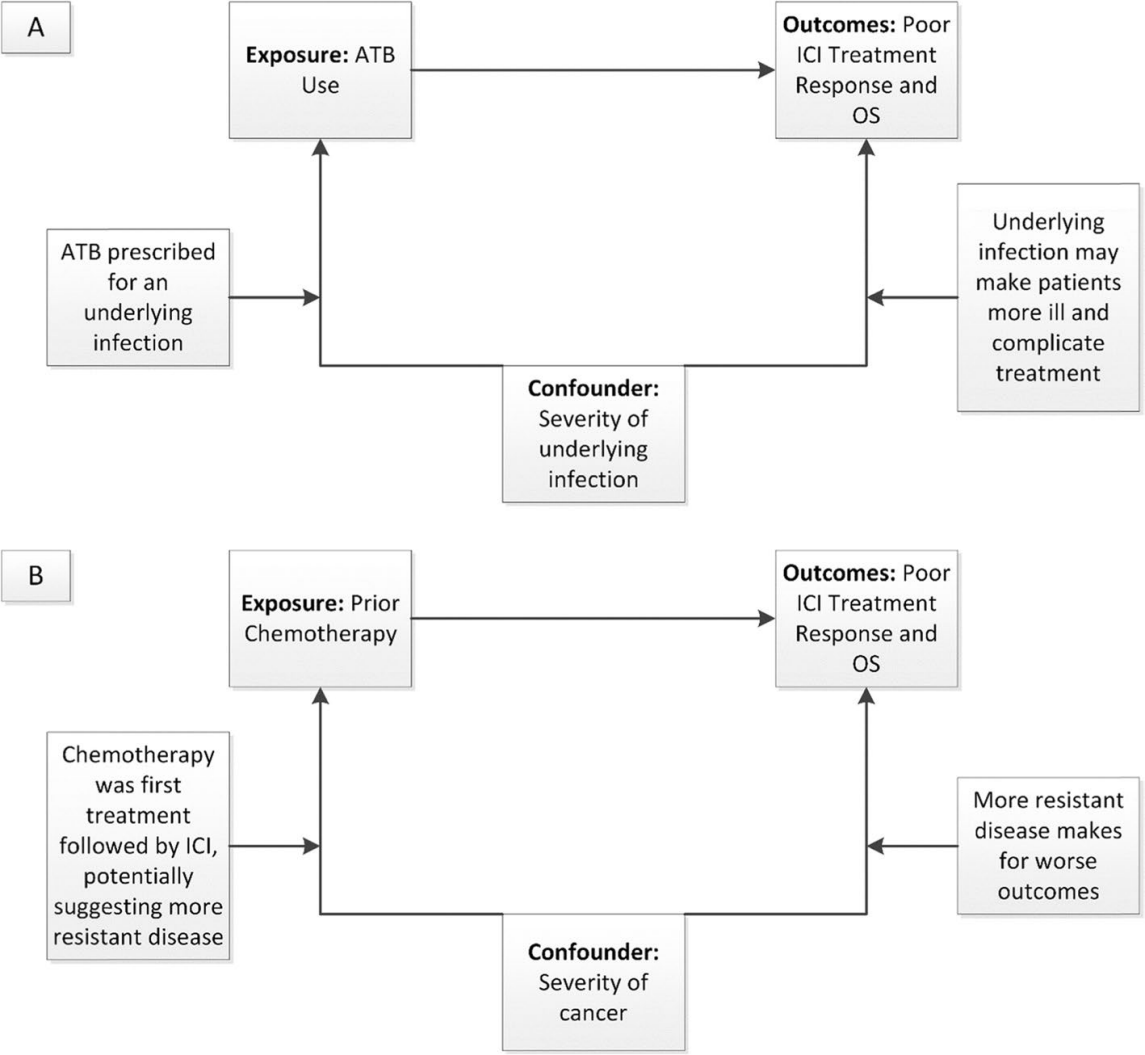
Example of confounding by indication. Confounding factors are variables associated with both the exposure and outcome of interest (i.e., poor treatment response and OS). **A** In this study, the reason for ATB use may act as a confounder, whereby an underlying infection, for example, may be indicative of a poorer health status at the start of ICI treatment, predisposing those individuals on ATBs to worse outcomes. **B** Similarly, having received previous chemotherapy treatment might indicate an inherently more resistant disease phenotype by the start of ICI treatment, predisposing those who had prior chemotherapy to worse outcomes. However, it should also be noted that chemotherapy was the only systemic treatment available as the standard of care for patients from 2011–2015

An important limitation of this retrospective study is the potential impact of confounding by indication [34] (Fig. 2). The association between ATB use and diminished OS identified in this study may be confounded by infectious comorbidity, as has been suggested in other studies of ATB use in patients treated with ICIs [19, 23, 35]. While 33% of patients only had one or two doses of prophylactic perioperative ATBs for surgery, the other 67%, who were treated for respiratory infections or for other reasons, may have had poorer health than patients who did not require ATBs and hence, were predisposed to have worse ICI response. Nonetheless, we did not find an association between the reason for ATB use and response, and we controlled for certain co-morbidities in this study using the CCI and for patient functionality via ECOG performance status, neither of which differed significantly between ATB users and non-users, unlike in previous research studies [36]. It is also possible that concomitant use of corticosteroids and other factors not measured in this study but associated with overall survival in cancer patients, like body mass index, may have confounded the association between ATB use and outcomes [37]. Finally, our study did not document other lifestyle factors that are known to modify the gut microbiome, including diet, exercise, pro- and prebiotics [38], as these were not available retrospectively in the chart review.

Another limitation of this study relates to sample size. Although we retrospectively analyzed a large number of patients over an extended period of time, only 46 received ATBs during the defined time period. Nevertheless, exposure misclassification, that is, excluding patients receiving undocumented ATBs from outside healthcare providers, may have influenced the statistical significance of the relationship observed between ATB use and poor ICI outcomes. Also, 33% of ATB users received only a prophylactic, single dose of ATBs prior to surgery. The effect of a very transient ATB exposure on the gut microbiome is unclear and may have biased results towards the null in this study. Future research should therefore investigate the association between antibiotic use and treatment outcomes in cancer patient cohorts comprised of higher proportions of antibiotic users, preferably in a prospective study for more accurate accounting of antibiotic use.

Although this study addresses ATB use two months before to one month after the start of ICI, it is possible that ATB use during the longer course of immunotherapy treatment may influence clinical outcomes. A recent multicenter study evaluated the influence of both prior and concurrent ATB use on ICI response, but found no significant association between concurrent ATB use and treatment response [23]. Other recent meta-analyses have determined that ATB use within 1–2 months *prior* to ICI treatment start and within 1–2 months *after* ICI start resulted in poorer outcomes compared to ATB use at more distant periods of time, highlighting the role of the gut microbiome in priming the immune system and how ATB exposure during an as yet undefined critical priming period may dampen ICI response [19, 20, 35]. Prospective cohort studies are needed to further study associations of concurrent ATB therapy and ICI treatment outcome to better characterize this critical window.

The finding that prior chemotherapy results in worse ICI response and OS is noteworthy. Although the negative effect of chemotherapy in ICI response may be related to the development of dysbiosis of the gut microbiome, there is also the possibility that these chemotherapy patients may have had intrinsically more resistant disease that may not have responded as well to ICI treatment (Fig. 2). However, during the time of the chart review from 2011–2017, ICIs were only available as standard of care off study after FDA approval of nivolumab in 2015 [39], so all stage IV NSCLC patients prior to 2015 routinely received the only available treatment of chemotherapy, which is rarely curative, leading to inevitable relapse. Then, following disease progression, they were treated on study (or off study after 2015) with ICIs. Thus, it is unlikely that more resistant disease accounts for the inferior ICI response observed in this study in patients with prior chemotherapy.

## Conclusions

Our study suggests that patients who receive ATBs near the time of ICI initiation may be more likely to experience poor outcomes than unexposed patients, although differences did not reach statistical significance, warranting further prospective exploration of this association. Prior chemotherapy, on the other hand, resulted in a significant negative impact on ICI treatment response and survival. The current study utilized ATBs and chemotherapy as a proxy for microbial dysbiosis to hypothesize the importance of an eubiotic gut microbiome in mediating immunotherapy efficacy in NSCLC.

Several recent studies suggest that specific compositions of bacteria in the gut microbiome are responsible for driving differential ICI outcomes in patients, although the mechanisms underlying these associations remain largely unknown. In order to investigate whether alterations in the gut microbiome are responsible for mediating ICI efficacy, our research team is beginning a large, prospective study of treatment response and the gut microbiome using metagenomic analyses of prospectively collected stool and blood samples from ICI responders and non-responders, including metabolomics, immune measures, and clinical data to more definitively answer these important questions.

## Data Availability

The data in this present study contain confidential patient medical information protected by HIPPA law from public view. It is maintained in a password-protected database and not publicly available. The datasets analyzed during the current study are available in a de-identified format from the corresponding author upon reasonable request.

## Abbreviations

ATB(s): Antibiotic(s)
CCI: Charlson Comorbidity Index
CR: Complete Response
CTLA-4: Cytotoxic T-lymphocyte-Associated Protein 4
ECOG: Eastern Cooperative Oncology Group
FMT: Fecal Microbiota Transfer
ICI: Immune Checkpoint Inhibitor
MCC: Moffitt Cancer Center
NSAIDs: Non-Steroidal Anti-Inflammatory Drugs
NSCLC: Non-Small Cell Lung Cancer
OS: Overall Survival
PD: Progressive Disease
PD-1: Programmed Cell Death-1
PD-L1: Programmed Death Ligand-1
PPIs: Proton-Pump Inhibitor
PR: Partial Response
PS: Performance status
RECIST: Response Evaluation Criteria in Solid Tumors
SD: Stable Disease
